# Endoscopic Ultrasound-Guided Radiofrequency Ablation of Metastatic Pancreatic VIPoma: A Novel Treatment

**DOI:** 10.7759/cureus.67114

**Published:** 2024-08-18

**Authors:** Sundeep Lakhtakia, Kritin Mehrotra, Anuradha Sekaran, Srivenu Itha, Nageshwar Reddy Duvvur

**Affiliations:** 1 Department of Gastroenterology and Hepatology, Asian Institute of Gastroenterology, Hyderabad, IND; 2 Internal Medicine, Armed Forces Medical College, Hyderabad, IND; 3 Department of Pathology, Asian Institute of Gastroenterology, Hyderabad, IND; 4 Gastroenterology, Cygnus Institute of Gastroenterology, Hyderabad, IND

**Keywords:** pancreatic neuroendocrine tumors, endoscopic ultrasound, radiofrequency ablation, men 1 syndrome, metastatic vipoma

## Abstract

VIPomas are a rare type of functional pancreatic neuroendocrine tumors (PNETs), causing symptoms due to their hypersecretion in the gastrointestinal tract. The rare association of PNETs with tumors of endocrine organs such as pituitary and parathyroid glands is called multiple endocrine neoplasia (MEN1) syndrome. Due to their indolent course, VIPomas often present late in the illness and may already have metastatic disease. The index case had MEN1 syndrome with biopsy-proven small sub-centimetric metastatic VIPoma and a history of parathyroidectomy for nodules in the past. The patient had a suspicion of pancreatic cholera and, after an appropriate workup, was treated by endoscopic ultrasound (EUS)-guided radiofrequency ablation (RFA) of the metastatic pancreatic VIPomas. EUS-guided RFA is a procedure by which the lesions are viewed under endoscopic ultrasound and undergo coagulative necrosis due to the high temperatures the tissue is subjected to. The application of EUS-guided radiofrequency ablation for sub-centimetric metastatic pancreatic VIPoma as a daycare procedure can be a valuable tool in its management.

## Introduction

Vasoactive intestinal peptide (VIP) is a gastrointestinal peptide discovered by Gardner and Cerda [1], demonstrating its action primarily, but not exclusively, along the gastrointestinal tract. It acts by stimulating adenosine 3’, 5’ cyclic phosphate (AMP) production by the intestinal tract, leading to profuse, explosive watery diarrhea and associated electrolyte imbalance, with potassium loss being the most commonly detected abnormality [2]. It has also been documented that VIP affects the respiratory system, growth and carcinogenesis, and the immune system, and is also known to have vaso-cardiac and neuronal effects [1]. The classical presentation of VIPoma involves the triad of profuse, watery diarrhea, hypokalaemia, and achlorhydria (WDHA syndrome or pancreatic cholera), but it may also present with weight loss, dehydration, hypercalcemia, hyperglycemia, nausea, and vomiting. Rarely, the patients may present to the clinic with complaints of generalized weakness, skin lesions, acute renal failure, acute flaccid hemiparesis, or paraparesis [3]. The majority of the lesions are detected within the pancreatic body and tail (75%), while 25% are encountered in the head of the pancreas. Extra pancreatic sites, although extremely rare, such as the bronchus, colon, liver, and neural crest-derived structures such as sympathetic chains, pituitary, thyroid, and adrenal, have also been reported in the literature [2].

Most of the cases of PNETs are isolated tumors, but in less than 5% of the cases, they can be associated with tumors of other endocrine organs in the body, such as the pituitary and parathyroid, known as Multiple Endocrine Neoplasia 1 syndrome, or MEN1 syndrome [4]. The diagnosis of MEN1 syndrome can be made clinically by showing the presence of neoplastic disease in at least two of the most commonly affected organs, namely the parathyroid, pituitary, or pancreas [5].

The present study describes the case of a 64-year-old female who presented to the hospital with complaints of chronic diarrhea for over two years, had a history of parathyroidectomy 15 years ago, and was currently diagnosed to have a pancreatic VIPoma with metastases to the paraduodenal lymph nodes.

## Case presentation

A 64-year-old female presented to the hospital with complaints of chronic watery diarrhea lasting for over two years, for which she had been undergoing symptomatic treatment at a different local hospital. After getting no relief despite various medications, she came to our hospital for further evaluation. She also gave a history of surgery done for the removal of a parathyroid nodule 15 years ago. The basic blood investigations were normal except for hypercalcemia (10.9 mg/dL, the normal range being 8.5-10.2 mg/dL), which was noted. A contrast-enhanced CT scan of her abdomen revealed no lesions in her pancreas or GI tract. Upper GI endoscopy was also done, which showed gastric body diminutive polyps and edematous mucosa with signs of duodenitis. Biopsies were taken, which revealed Brunner’s gland hyperplasia and peptic duodenitis. A colonoscopy was also done, which was non-contributory. She was advised symptomatic treatment but had no relief in symptoms even after two months.

Laboratory examination for free metanephrine and nor-metanephrine levels was normal, with mildly raised serum prolactin (32.6 ng/mL, normal range being 4.79-23.3 ng/mL) and Gastrin (424.0 pg/mL, normal range being 13.0-115.0 pg/mL) levels. The rise in her gastrin levels was attributed to her use of proton pump inhibitors. She was later admitted to the hospital, and her Ga68 DOTANOC PET CT scan showed a somatostatin-expressing, small hypodense, mildly enhancing tumor in the distal body and tail of the pancreas, along with increased isotope uptake in the first part of the duodenum and enhancing paraduodenal lymph nodes indicative of a metastatic neuroendocrine tumor. Medullary calcinosis of the right kidney was also seen, consistent with a history of parathyroid adenoma and findings suggestive of MEN1 syndrome. The patient was given a subcutaneous injection of Somatostatin 30 mg after the PET CT and reported dramatic relief in symptoms. Plasma VIP levels were in the normal range, and serum chromogranin levels were markedly elevated at 1360 ng/mL (Table 1).

**Table 1:** The measured lab values of the patient

Test	Value	Normal Range
Gastrin	424 pg/mL	13-115 pg/mL
Prolactin	32.6 ng/mL	4.79-23.3 ng/mL
Calcium	10.9 mg/dL	8.5-10.2 mg/dL
Chromogranin	1360 ng/mL	< 39 ng/mL
Free Metanephrine	32 pg/mL	< 100 pg/mL
Free Nor-Metanephrine	185 pg/mL	< 216 pg/mL

**Figure 1:**
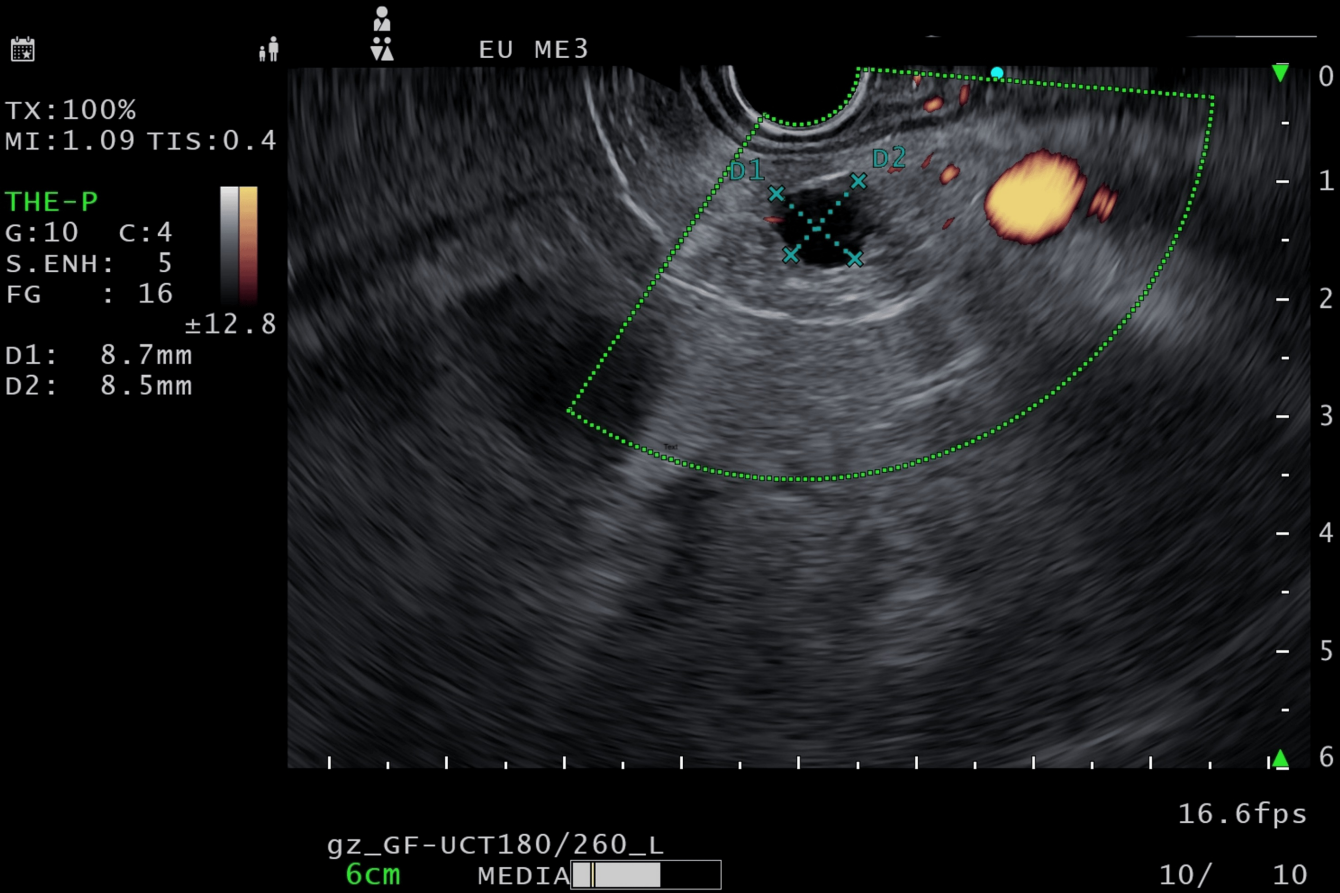
EUS localization of VIPoma EUS: Endoscopic ultrasound

**Figure 2:**
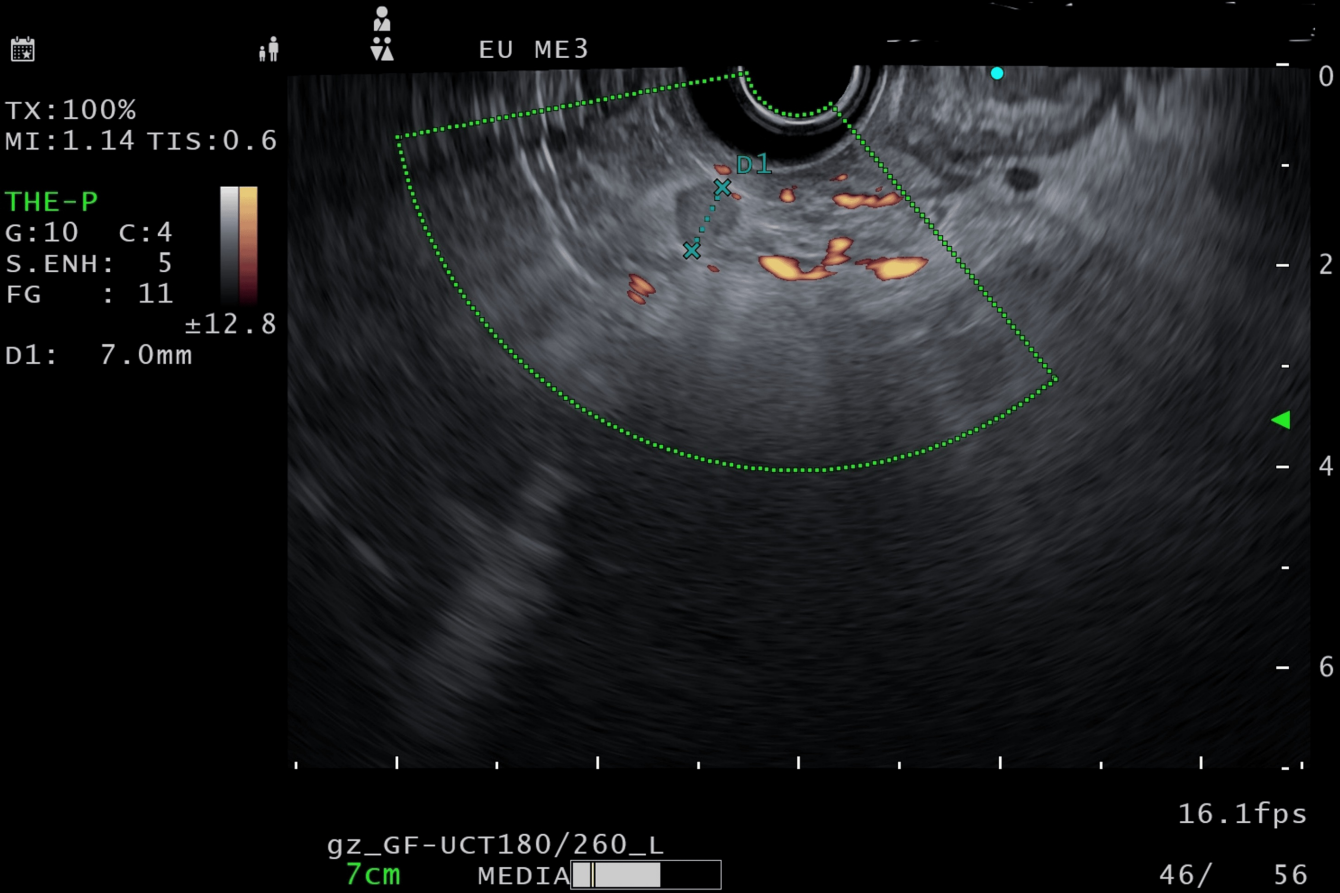
EUS localization of metastatic paraduodenal lymph nodes EUS: Endoscopic ultrasound

An endoscopic ultrasound (EUS) was done, which demonstrated multiple small hypoechoic lesions in the body of the pancreas, <5 mm in size, with the largest being cystic, with well-defined borders about 8.7 x 8.5 mm in the body of the pancreas, a mildly thickened wall, and no contrast enhancement on Doppler or direct communication with the pancreatic duct (Figure 1). A few small paraduodenal lymph nodes were slightly enlarged in size, the largest being 7 mm in size, without increased vascularity or irregular borders (Figure 2). Liver echotexture was normal, without any metastatic lesions. An EUS-guided fine needle biopsy of the largest lesion, measuring 8.7x8.5 mm, was done using a 22G core needle, and samples were sent for a core biopsy and cytology to confirm the diagnosis. Immediate refilling of the cystic lesion post-FNB was observed.

Histopathology of the specimen revealed a few monomorphic cell clusters arranged in nests, suspicious for a neuroendocrine lesion. Immunohistochemistry analysis was positive for synaptophysin and chromogranin but CD45 (-) and Ki67 0-1%, thus confirming a well-differentiated Grade 1 neuroendocrine tumor.

**Figure 3:**
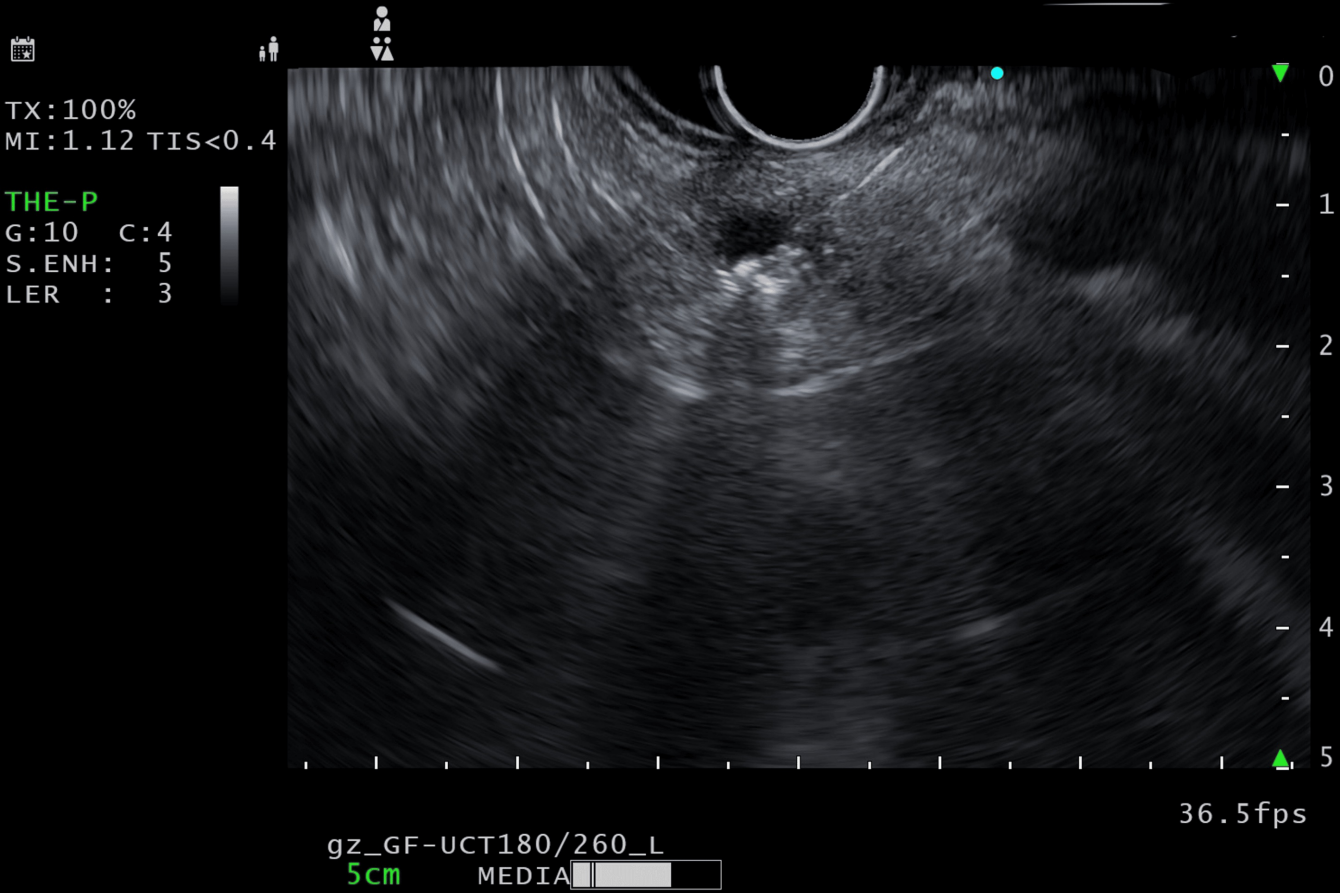
The EUSRA needle was introduced into the lesion EUSRA: Endoscopic ultrasound radiofrequency ablation

**Figure 4:**
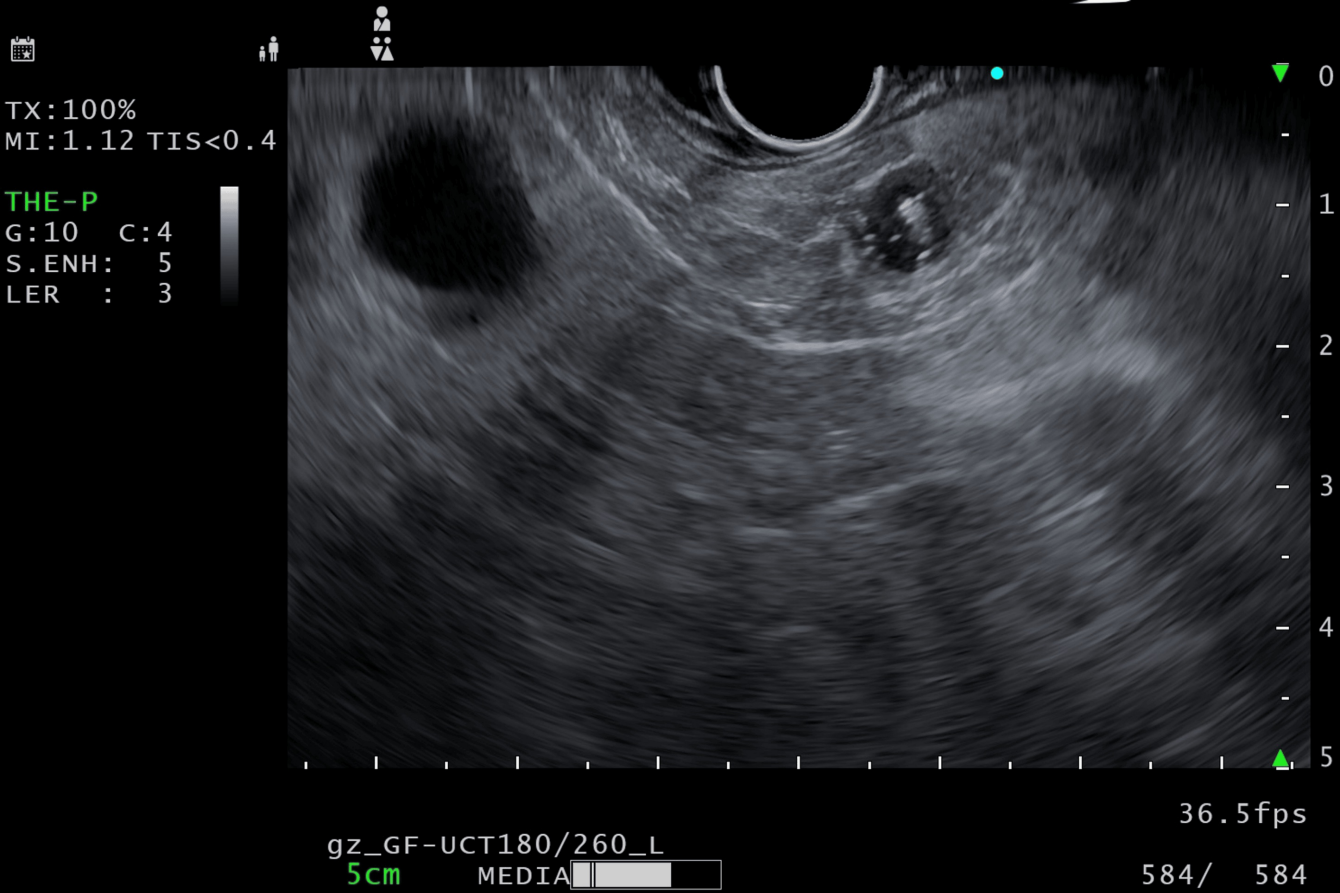
Post-procedural EUS EUS: Endoscopic ultrasound

Two weeks after the histopathological assessment, EUS-guided radiofrequency ablation (EUSRA) of the pancreatic cystic NET was done via the stomach under monitored anesthesia care using an EUSRA needle at 30 W (Figures 3, 4), and two sites were ablated. The intra- and post-procedural period was uneventful, and she was discharged within 24 hours. 

## Discussion

Pancreatic VIP-secreting tumors are a rare group of islet cell tumors, with their incidence being recorded at one per 10 million individuals annually [4]. These tumors manifest in the fourth to sixth decade of life, whereas children may also develop these tumors due to genetic predisposition [2]. While the classical presentation of VIPoma involves the triad of profuse, watery diarrhea, hypokalemia, and achlorhydria (WDHA syndrome or pancreatic cholera), patients may also present with hypercalcemia, hyperglycemia, dehydration, and flushing [3]. We report a rare combination of a middle-aged female with pancreatic VIPoma and MEN1 instead of gastrinoma, which is by far a more common association with MEN1. The true incidence of VIPoma in patients with MEN1 is probably not known, as it has only been reported in a few patients.

Laboratory analysis and imaging play an important role in the diagnosis of VIPoma. Plasma VIP levels >200 pg/mL are the accepted standard concentration for diagnosing VIPoma [6]. Serum chromogranin A levels are also elevated and are useful in estimating the tumor burden [7]. The index case had markedly elevated serum chromogranin levels at 1360 ng/mL but, on the contrary, had normal VIP levels, possibly due to the injection of somatostatin that she received before the collection of the sample. Timothy et al. have also described in their pathophysiology of somatostatin that it has an inhibitory effect on the secretion of VIP [8]. Elevated VIP levels were seen in only 73.3% of patients in the study by Amiri [3].

Imaging plays a vital role in confirming the diagnosis, choosing the modality of treatment, and determining the prognosis of the patient. The initial imaging of choice for VIPomas is a helical multiphase contrast-enhanced CT/MRI scan of the abdomen. Gallium 68 DOTATATE is a PET radiotracer that is a form of somatostatin-receptor functional imaging used for the evaluation of primary and metastatic well-differentiated neuroendocrine tumors. The sensitivity and specificity of Ga 68 DOTATATE PET/CT are reported to be 90% and 90.6%, respectively, and even higher, approaching 100% in some studies. In our case, it played a significant role in the localization of the tumor along with identifying the metastatic spread when contrast CT was normal [9]. We recommend the use of Ga68-DOTATATE PET/CT in negative CT/MRI scans with elevated tumor marker levels. The higher cost and limited availability are big limiting factors for its usage.

Treatment options include symptomatic therapy, chemotherapy, radiation, and locoregional therapies like chemoembolization, radiofrequency ablation, and surgery [10]. The first line of treatment for VIPomas is surgical excision for patients with benign and non-metastatic disease, with no accepted standard of management for patients with metastatic disease [6]. Surgical options for metastatic VIPoma include surgery, with distal pancreatectomy with or without splenectomy being the most common, followed by Whipple’s procedure, and each of them can have serious complications. Chemotherapy includes the doxorubicin/streptozotocin combination as the gold standard, with 5-fluorouracil replacing doxorubicin if it’s contraindicated [4]. Somatostatin analogs (SSAs) such as octreotide and lanreotide are used for symptomatic relief and inhibition of tumor growth [2]. The application of radiofrequency ablation for sub-centimetric metastatic VIPoma as a daycare procedure can be a valuable tool in the management of VIPomas, and due to its low incidence, there are no prospective studies or evidence-based therapeutic standards to date.

## Conclusions

The possibility of pancreatic VIPoma should be considered in a patient with persistent diarrhea associated with dyselectroytemia. We recommend the use of Ga68-DOTATATE PET/CT in negative CT/MRI scans with elevated tumor marker levels. The application of radiofrequency ablation for sub-centimetric metastatic VIPoma as a daycare procedure can be a valuable tool in the management of VIPomas.
